# Células Progenitoras Endoteliais e Exercício: Trabalhando Juntos Contra a Disfunção Endotelial na Síndrome Metabólica

**DOI:** 10.36660/abc.20210399

**Published:** 2021-07-15

**Authors:** Christina Grüne de Souza e Silva

**Affiliations:** 1 Clínica de Medicina do ExercícioRio de JaneiroRJBrasilClínica de Medicina do Exercício – CLINIMEX,Rio de Janeiro, RJ - Brasil

**Keywords:** Atividade Física, Função Endotelial, Óxido Nítrico, Doença Cardiovascular, Síndrome Metabólica

Nossa compreensão do papel crucial desempenhado pelo endotélio na biologia cardiovascular evoluiu nas últimas duas décadas, com o reconhecimento de que é um órgão regulado dinamicamente, essencial na manutenção da homeostase vascular. Um endotélio saudável é capaz de responder adequadamente a sinais físicos e químicos e, através da liberação de uma ampla gama de mediadores, regular, entre outros, o tônus e o crescimento vascular, a adesão celular, a trombogenicidade e a inflamação.^1^

Além disso, já foi reconhecido que quando o endotélio perde suas propriedades fisiológicas devido a um desequilíbrio entre sua lesão e sua recuperação adequada, há uma tendência a estados de vasoconstrição, pró-trombóticos e pró-inflamatórios. Esta condição, chamada de disfunção endotelial, demonstrou preceder o desenvolvimento de alterações pró-ateroscleróticas, levando à formação de placa aterosclerótica e suas complicações clínicas posteriores.^2^

As células progenitoras endoteliais (CPE) são um subtipo de células derivadas da medula óssea que expressam marcadores endoteliais e progenitores e que são mobilizadas ou liberadas na circulação sistêmica em resposta a estímulos específicos, contribuindo para a formação de vasos e para o reparo endotelial.^3^ Um grupo crescente de evidências mostraram uma correlação inversa entre a atividade funcional das CPE e os fatores de risco cardiovascular (CV) e, portanto, não apenas as CPE foram consideradas um indicador independente da saúde CV em geral, mas também um potencial alvo terapêutico em condições de alto risco CV.^4^

A síndrome metabólica (SM), um importante precursor da doença CV, tem sido considerada um dos principais contribuintes para uma fraca funcionalidade das CPE.^5^ A resistência à insulina, a hiperglicemia, a obesidade, a dislipidemia e a hipertensão agem através de uma variedade de vias diferentes que, por fim, prejudicam a mobilização, proliferação e sobrevivência das CPE.^6^

Ao contrário, o exercício físico tem consistentemente demonstrado melhorar a função das CPE, contribuindo para a promoção e manutenção de um endotélio saudável e para uma redução de eventos CV.^7,8^ Portanto, as diretrizes atuais sobre prevenção CV reconhecem e enfatizam a prática regular de exercício físico como uma estratégia-chave no manejo da SM.^9^ Entretanto, embora haja um interesse crescente em explorar os benefícios induzidos pelo exercício nas CPE nessa população de alto risco, seus mecanismos moleculares estão longe de ser totalmente compreendidos.

Nesta edição dos Arquivos Brasileiros de Cardiologia, Tan et al.^10^ compararam a funcionalidade das CPE em 30 indivíduos fisicamente inativos com SM que foram aleatoriamente designados a um grupo exercício (n = 15) ou a um grupo controle (n = 15). Após oito semanas de participação em sessões regulares de exercícios que incluíam exercícios aeróbicos de média intensidade e exercícios não-aeróbicos, os indivíduos do grupo exercício mostraram melhora da função das CPE, expressa por uma capacidade aumentada de gerar unidades formadoras de colônias de células endoteliais e melhora na formação de tubos. Além disso, os autores foram capazes de identificar uma maior ativação do eixo de sinalização PI3K-Akt-eNOS no grupo exercício, o que pode contribuir, pelo menos em parte, para a manutenção da homeostase cardiovascular e integridade dos vasos.^11^

É importante notar que, embora haja um número limitado de estudos comparando resultados clínicos de acordo com diferentes intervenções de exercício no cenário da SM, treinamento de média intesidade vs. de alta intensidade e exercícios aeróbicos vs. exercícios resistidos, realizados em combinação ou isoladamente, parecem ter benefícios diferentes sobre os componentes da SM.^12^ Assim, é razoável especular que os achados positivos na função das CPE relatados por Tan et al.,^10^ podem não ser generalizáveis para outros programas de exercício.

Portanto, o estudo de Tan et al.,^10^ fornece novas evidências que apoiam o conceito de que o exercício físico tem um efeito benéfico na capacidade de reparo endotelial das CPE na SM. Ainda assim, estudos futuros são necessários para avaliar se diferentes modalidades de exercício físico têm efeitos diferentes sobre as CPE, a fim de permitir a prescrição eficiente de exercícios para indivíduos com esse complexo grupo de distúrbios metabólicos altamente prevalente.
