# Supplementary material for: Células Progenitoras Endoteliais e Exercício: Trabalhando Juntos Contra a Disfunção Endotelial na Síndrome Metabólica
Source: Arq Bras Cardiol. 2021 Jul 15;117(1):118–9. [Article in Portuguese] doi: 10.36660/abc.20210399 (PMC8294726; doi:10.36660/abc.20210399)
Supplement: Translation

# Translation: Endothelial Progenitor Cells and Exercise: Working Together to Target Endothelial Dysfunction in Metabolic Syndrome

**Authors:** Christina Grüne de Souza e Silva

**Affiliations:** 1 BrazilExercise Medicine Clinic – CLINIMEX, Rio de Janeiro, RJ - Brazil

**Keywords:** Physical Activity, Endothelial Function, Nitric Oxide, Cardiovascular Disease, Metabolic Syndrome

Our understanding of the crucial role played by the endothelium in cardiovascular biology has evolved over the past couple of decades, with the recognition that it is a dynamically-regulated organ, essential in maintaining vascular homeostasis. A healthy endothelium is able to adequately respond to physical and chemical signals and, through the release of a wide range of mediators, regulate, among others, vascular tone and growth, cellular adhesion, thrombogenicity, and inflammation.^1^

Furthermore, it has been acknowledged that when the endothelium loses its physiological properties due to an imbalance between its injury and its appropriate recovery, a tendency towards vasoconstriction, pro-thrombotic, and pro-inflammatory states are shown. This condition, termed endothelial dysfunction, has been shown to precede the development of pro-atherosclerotic changes, leading to atherosclerotic plaque formation and its later clinical complications.^2^

Endothelial progenitor cells (EPCs) are a subtype of bone marrow-derived cells that express both endothelial and progenitor markers and that are mobilized or released into systemic circulation in response to specific stimuli, contributing to vessel formation and endothelial reparation.^3^ A growing body of evidence has shown an inverse correlation between the functional activity of EPCs and cardiovascular (CV) risk factors, and, therefore, not only EPCs have been considered an independent indicator of overall CV health, but also a potential target for therapeutics in high CV risk conditions.^4^

Metabolic syndrome (MS), an important precursor of CV disease, has been implied as a major contributor to a weak functionality of EPCs.^5^ Insulin resistance, hyperglycemia, obesity, dyslipidemia and hypertension act through a number of different pathways that ultimately impairs EPCs’ mobilization, proliferation, and survival.^6^

Conversely, physical exercise has been consistently shown to improve EPC function, contributing to the promotion and maintenance of a healthy endothelium, and to a reduction in CV events.^7,8^ Therefore, current guidelines on CV prevention have acknowledged and underscored the engagement in regular physical exercise as a key management strategy for MS.^9^ However, although there is a growing interest in exploring the exercise-induced benefits on EPCs in this high risk population, its molecular mechanisms are far from being fully understood.

In this issue of the *Arquivos Brasileiros de Cardiologia*, Tan et al.^10^ compared the functionality of EPCs in 30 physically inactive individuals with MS who were randomly assigned to an exercise group (n=15) or to a control group (n=15). After eight weeks of participation in regular exercise sessions that included both moderate-intensity aerobic and non-aerobic exercises, individuals from the exercise group showed enhanced EPC function expressed by an increased capacity to generate endothelial cell-colony forming units and enhanced tube formation. Moreover, the authors were able to identify a higher activation of the PI3K-Akt-eNOS signaling axis in the exercise group, which might contribute, at least in part, to the maintenance of cardiovascular homeostasis and vessel integrity.^11^

It is important to note that, even though there is a limited number of studies comparing clinical outcomes according to different exercise interventions in the setting of MS, moderate vs. high-intensity training and aerobic vs. resistance exercise, either performed in combination or alone, seem to have different benefits on the MS components.^12^ As such, it is reasonable to speculate that the positive findings in EPC function reported by Tan et al.^10^ might not be generalizable to other exercise training programs.

Therefore, the study of Tan et al.^10^ provides novel evidence that support the concept that physical exercise exerts a beneficial effect on endothelial repair capacity of EPCs in MS. Yet, future studies are needed to evaluate if different modalities of physical exercise have different effects on EPCs in order to allow efficient exercise prescription for individuals with this highly prevalent and complex cluster of metabolic disturbances.
